# Art, neuroplasticity, and tradition: a narrative review and conceptual perspective of Chinese woodblock printing in global art practices and Parkinson’s disease neuroscience

**DOI:** 10.3389/fnins.2026.1853378

**Published:** 2026-08-21

**Authors:** Kailun Lin, Qi Fang, Kun Li

**Affiliations:** 1 College of Arts, Chengdu Sport University, Chengdu, Sichuang, China; 2 School of Art and Design, Weifang University of Science and Technology, Shouguang, Shandong, China

**Keywords:** art therapy, Chinese woodblock printing, neuroplasticity, Parkinson’s disease, rehabilitation, sensorimotor engagement, traditional arts, visuospatial processing

## Abstract

Parkinson’s disease (PD) is a progressive neurodegenerative disorder characterized by debilitating motor impairment, cognitive decline, and a substantial reduction in patient quality of life. While pharmacological treatments and standard physiotherapy provide essential symptomatic relief, they often offer only transient benefits. Consequently, there is growing interest in non-pharmacological interventions that may support sustained improvements in motor function, balance, and emotional well-being through neuroplastic mechanisms. This narrative review and conceptual perspective synthesizes evidence linking creative art practices to neuroplasticity and develops a hypothesis-generating framework for the potential relevance of traditional Chinese woodblock printing as a culturally grounded sensorimotor activity in PD rehabilitation. Literature across four thematic domains, art therapy in Parkinson’s disease, fine-motor and sensorimotor rehabilitation, neuroplasticity mechanisms, and culturally grounded creative interventions, was surveyed across PubMed, Scopus, and Google Scholar; no primary empirical studies evaluating Chinese woodblock printing in any neurological rehabilitation population were identified. However, review-level evidence and individual studies of related fine-motor modalities, including calligraphy, drawing, painting, rhythmic drumming, and visual art therapy, consistently report improvements in motor severity, balance, fine-motor control, and psychosocial outcomes. These therapeutic gains are attributed to repetitive fine-motor engagement, multisensory integration, and the strengthening of fronto-parieto-motor networks, supported by experience-dependent cortical reorganization. Chinese woodblock printing integrates precision carving, rhythmic inking, and manual printing, demanding complex bilateral coordination, visuospatial planning, and executive sequencing. The proposed conceptual framework posits that sustained printmaking practice drives intensive sensorimotor engagement, leading to functional gains in motor control and manual dexterity. Although direct empirical evidence remains absent, the biomechanical and neural parallels with established interventions suggest that woodblock printing represents a promising, low-cost adjunctive neurorehabilitation tool. This review highlights the need for modality-specific randomized controlled trials and longitudinal neuroimaging data to test the hypotheses generated by culturally resonant arts in Parkinson’s disease care. Integrating such practices into community-based rehabilitation may offer scalable, holistic benefits that complement conventional medical therapies.

## 1 Introduction

Parkinson’s disease (PD) is a progressive neurodegenerative disorder characterized by motor impairment, cognitive decline, and diminished quality of life. Global epidemiological data from large systematic reviews and burden-of-disease analyses (2020–2024) document a rising prevalence and mortality, together with marked sex and age disparities (Zhu et al., 2024). A meta-analysis of 83 studies across 37 countries reported a pooled prevalence of 1.51 cases per 1,000 (95% CI 1.19–1.88), increasing from 0.90 per 1,000 (1980–1989) to 3.81 per 1,000 (2010–2023) (Zhang et al., 2021). In 2021, incidence reached 79.68 per 100,000 and disability-adjusted life years stood at 477.50 per 100,000; both metrics rose over 32 years (estimated annual percentage changes of 1.2 and 0.6%, respectively) (Lampropoulos et al., 2022). Mortality rates increased from 1.76 to 5.67 per 100,000 between 1994 and 2019, with male-to-female ratios of 1.18 for prevalence and 1.37 for incidence. Prevalence escalates sharply with age, reaching 9.34 per 1,000 in individuals older than 60 years (Pereira et al., 2024).

Given the limited long-term efficacy of pharmacological and standard physiotherapy interventions, there is increasing interest in non-pharmacological approaches to neurological rehabilitation. Among broader creative modalities, dance has shown benefit in some Parkinson’s disease studies, but the present review focuses on fine-motor arts with closer mechanistic alignment to printmaking, including calligraphy and visual art production. The six quality-of-life instruments with the strongest psychometric properties in PD are the PDQ-39, PDQ-8, PDQL, PDQUALIF, PIMS, and Neuro-QOL (Tomlinson et al., 2001). The CHAPO-PD model offers a holistic framework for longitudinal assessment that incorporates both challenges and resilience factors across disease progression (Okada et al., 2021).

Neuroplasticity, the brain’s ability to reorganize neural connections in response to learning and environmental stimulation, underpins the therapeutic potential of these approaches. Musical training induces coordinated functional and structural reorganization across sensorimotor and auditory networks in the adult brain. Functional changes include enhanced activation in premotor, supplementary motor, and primary motor cortices, strengthened connectivity within sensorimotor networks, and increased auditory-motor coupling via white-matter pathways such as the arcuate fasciculus (El Hayek et al., 2023). Structural reorganization of gray and white matter occurs within 6–15 months of training, with functional connectivity changes correlating positively with practice time (Chen et al., 2020). This evidence derives from multiple methodologies (fMRI, TMS, structural imaging) in controlled longitudinal and cross-sectional designs (Su et al., 2025; de la Cuadra-Grande et al., 2025).

Creative activities have been shown to enhance motor learning, sensory integration, and emotional regulation. A scoping review of 17 studies found that creative activities improved limb motor function, fine motor ability, and activities of daily living in stroke patients (Thieken et al., 2022). An RCT of 118 stroke patients demonstrated that creative art therapy combined with conventional physical therapy significantly enhanced physical function (mean difference 1.2, *p* = 0.043) compared with physical therapy alone (Herholz and Zatorre, 2012). Earlier work documented >20% gains in upper-extremity function and sensory discrimination after 12 h of supervised sensory-motor training (*p* < 0.01) (Olszewska et al., 2021). Structured creative activities also produced positive effects on motor planning, balance, and attention in a pediatric case of intellectual disability (Li et al., 2018).

Creative therapies exert modest to positive effects on emotional regulation in neurodegenerative populations. An RCT of 150 patients found that mind–body and art therapies reduced Difficulties in Emotion Regulation Scale scores by −4.8 versus −0.11 in controls, although the between-group difference was not statistically significant (*p* = 0.13) (Choi et al., 2015). Disease-specific studies, however, reported stronger outcomes: music therapy significantly improved emotional function on the Happiness Measure in 32 PD patients (*p* < 0.0001) (Barnett and Vasiu, 2024) and reduced behavioral/psychiatric symptoms on the Neuropsychiatric Inventory in 59 dementia patients (*p* = 0.002) (Strang, 2024). Recent pilot data in 8 PD patients confirmed significant anxiety reduction and well-being gains (Khalil and Demarin, 2023). Systematic reviews affirm effectiveness for behavioral and emotional symptoms, albeit with noted limitations in study quality and sample size (Kongkasuwan et al., 2016).

Traditional art practices may support neurological recovery through repetitive motor actions, visuospatial processing, and cognitive engagement. Culturally grounded practices involving repetitive motor actions, particularly calligraphy and drumming, have demonstrated meaningful improvements in motor recovery and control for both stroke and PD patients. Modified calligraphy exercise significantly improved upper-limb motor function on the Fugl-Meyer Assessment (*p* = 0.001) in stroke patients (Liu et al., 2024). A meta-analysis of 26 art-therapy studies in PD reported significant reductions in UPDRS III scores (SMD = −0.44, *p* < 0.05) and improved balance (Mini-BESTest SMD = 0.41, *p* < 0.05) (Byl et al., 2003). Drumming with rhythmic cueing enhanced upper-extremity motor control and attention, while case-series data showed favorable changes in balance and dexterity (Park and Kim, 2021).

Chinese woodblock printing is one such historic printmaking technique that combines artistic creativity with structured motor movements. No research has specifically examined neural activation during structured printmaking tasks or performed biomechanical analyses of Chinese woodblock printing techniques (Taghizadeh et al., 2018). The closest evidence derives from structured visuospatial tasks such as block design copying, which increased activation in memory-, motor-, and arithmetic-related regions (Llamas-Velasco et al., 2023). Broader visuospatial literature consistently identifies parietal (superior parietal lobule), prefrontal, and visual cortical involvement. These sensorimotor and cognitive demands suggest potential therapeutic benefits, yet direct evidence for woodblock printing remains absent and highlights a clear research gap (Ma et al., 2013).

Despite the growing body of research on art-based therapies, limited synthesis exists that links traditional printmaking practices to neuroscience and Parkinson’s disease rehabilitation. Existing literature on art-based interventions for Parkinson’s disease is constrained by methodological heterogeneity, small sample sizes, and a lack of standardized protocols (Ettinger et al., 2023; Spee et al., 2025). Key documented limitations include small samples (10–79 participants), absence of direct modality comparisons, inconsistent outcomes and comparators, male underrepresentation (55% in the largest review), and limited functional-communication data (Gómez-Soria et al., 2026). Nevertheless, art-based interventions integrated into neurological rehabilitation have shown promising effects compared with standard rehabilitation alone, improving motor function, cognitive abilities, and quality of life in controlled studies (Fodor et al., 2021; Machado Sotomayor et al., 2021).

**Figure 1:**
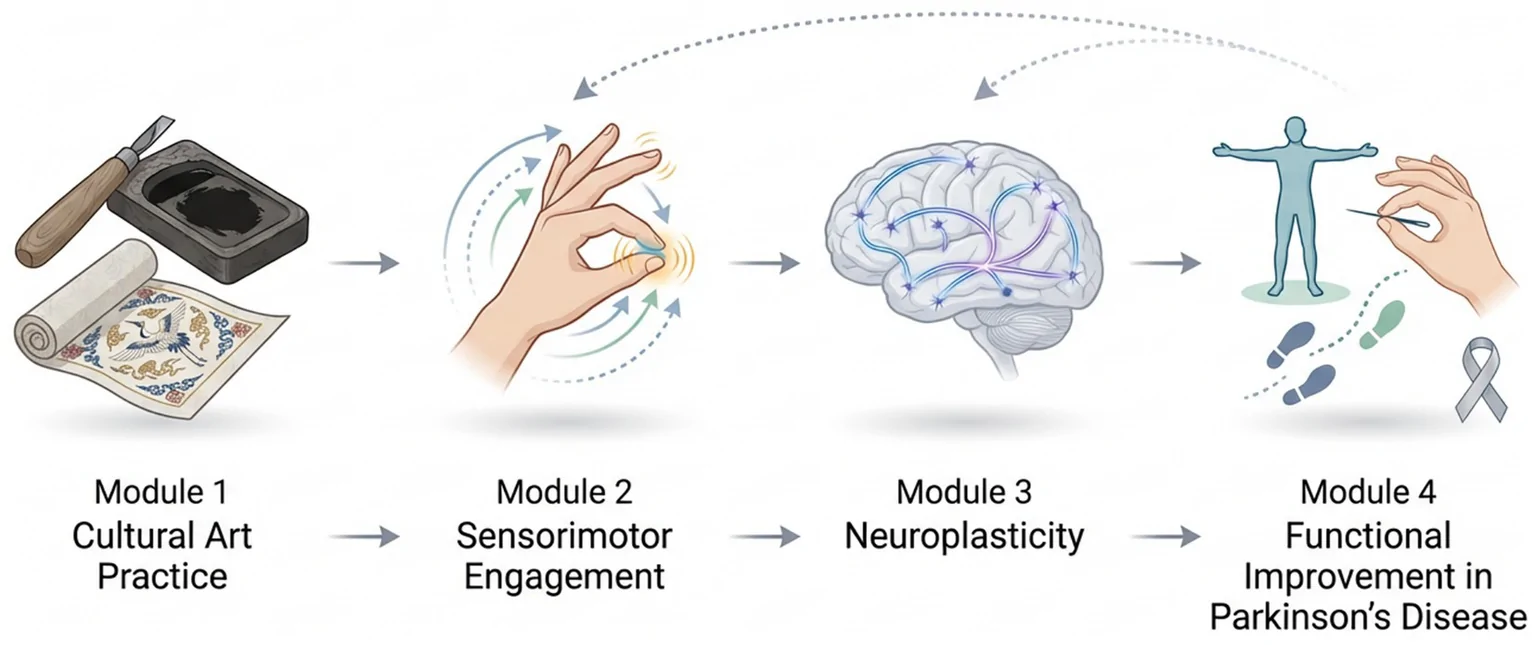
Proposed conceptual framework linking cultural art practice to functional outcomes in Parkinson’s disease. This schematic illustrates a hypothesized pathway in which engagement in traditional cultural art practices (e.g., Chinese woodblock printing) promotes sensorimotor engagement through coordinated, repetitive hand movements and multisensory input. Sustained engagement is proposed to drive neuroplastic changes within motor, premotor, and associated neural networks. These adaptations may translate into functional improvements in Parkinson’s disease, including enhanced motor control, balance, dexterity, and overall quality of life. The dotted feedback pathway indicates a potential reinforcing loop, whereby functional gains further support continued sensorimotor engagement and neural adaptation over time.

This manuscript is presented as a narrative review and conceptual perspective that explores the neurological and therapeutic potential of Chinese woodblock printing, examines related art-based approaches relevant to Parkinson’s disease, and develops a hypothesis-generating framework for future investigation. Rather than conducting a formal systematic review, this review synthesizes interdisciplinary literature on art therapy, fine-motor rehabilitation, neuroplasticity, and culturally grounded creative practice to develop a hypothesis-generating framework for Chinese woodblock printing in Parkinson’s disease. The overarching conceptual pathway through which cultural art practice may translate into functional outcomes in Parkinson’s disease is illustrated in Figure 1.

### 1.1 Narrative review scope

Literature for this review was identified through narrative searches of PubMed, Scopus, and Google Scholar conducted up to January 2026, covering four thematic domains: (I) art therapy and creative interventions in Parkinson’s disease; (II) fine-motor and sensorimotor rehabilitation in neurological populations; (III) neuroplasticity mechanisms underlying motor learning and creative practice; and (IV) culturally grounded rehabilitation approaches. Peer-reviewed studies and reviews were considered relevant when they reported neurological, motor, cognitive, or psychosocial outcomes of fine-motor, multisensory, or culturally tailored creative interventions in neurological populations, or when they provided mechanistic neuroplasticity evidence applicable to such interventions. Analog modalities, including calligraphy, rhythmic drumming, drawing, and visual art therapy, were selected on the basis of shared sensorimotor demands with Chinese woodblock printing, specifically sustained precision grip, bilateral hand coordination, visuospatial sequencing, and repetitive upper-limb engagement. A targeted search combining “Chinese woodblock printing” with “Parkinson’s disease,” “rehabilitation,” and “art therapy” returned no primary empirical studies, confirming the absence of direct clinical evidence and establishing the rationale for an analogy-based conceptual framework.

## 2 Historical and artistic foundations of Chinese woodblock printing

### 2.1 Origins and cultural significance

Chinese woodblock printing emerged in early imperial China during the Tang Dynasty (618–907 CE) and rapidly became a foundational technology for East Asian art and knowledge transmission. Scholars date its invention to approximately 600–700 CE, likely inspired by earlier practices of using bronze or stone seals for impressions on clay and silk, as well as inked rubbings of inscribed texts from stone reliefs (Barrett, 2001; Chia, 2020). By the end of the Tang period, the technique had been perfected, enabling the mass reproduction of texts and images on paper, an innovation that dramatically reduced costs and broadened access compared with labor-intensive hand-copying. The oldest dated example of woodblock printing is the Diamond Sutra (868 CE), a Buddhist text discovered in the Dunhuang Library Cave, which exemplifies the technology’s early application in disseminating religious and philosophical knowledge (Hansen, 2024; Huai-Dong et al., 2013).

During the subsequent Song Dynasty (960–1,279 CE), woodblock printing reached new heights of sophistication, supporting the production of Confucian classics, secular literature, medical texts, and illustrated works. This technological leap played a pivotal role in increasing literacy rates, fostering cultural exchange, and accelerating the spread of ideas across China and beyond. The technique diffused rapidly to Korea and Japan by the eighth century and later influenced printing traditions in Southeast Asia and the Middle East (Barrett, 2001; Korenberg et al., 2019). In East Asia, woodblock printing served not only as a practical tool for book production but also as a medium for artistic expression, including New Year prints, pictorial narratives, and political commentary. Its enduring cultural significance lies in its contribution to the preservation and global dissemination of knowledge, predating Gutenberg’s movable-type press by several centuries and shaping the intellectual landscapes of multiple civilizations (Chia, 2020; Song, 2009).

### 2.2 Technical process

**Figure 2:**
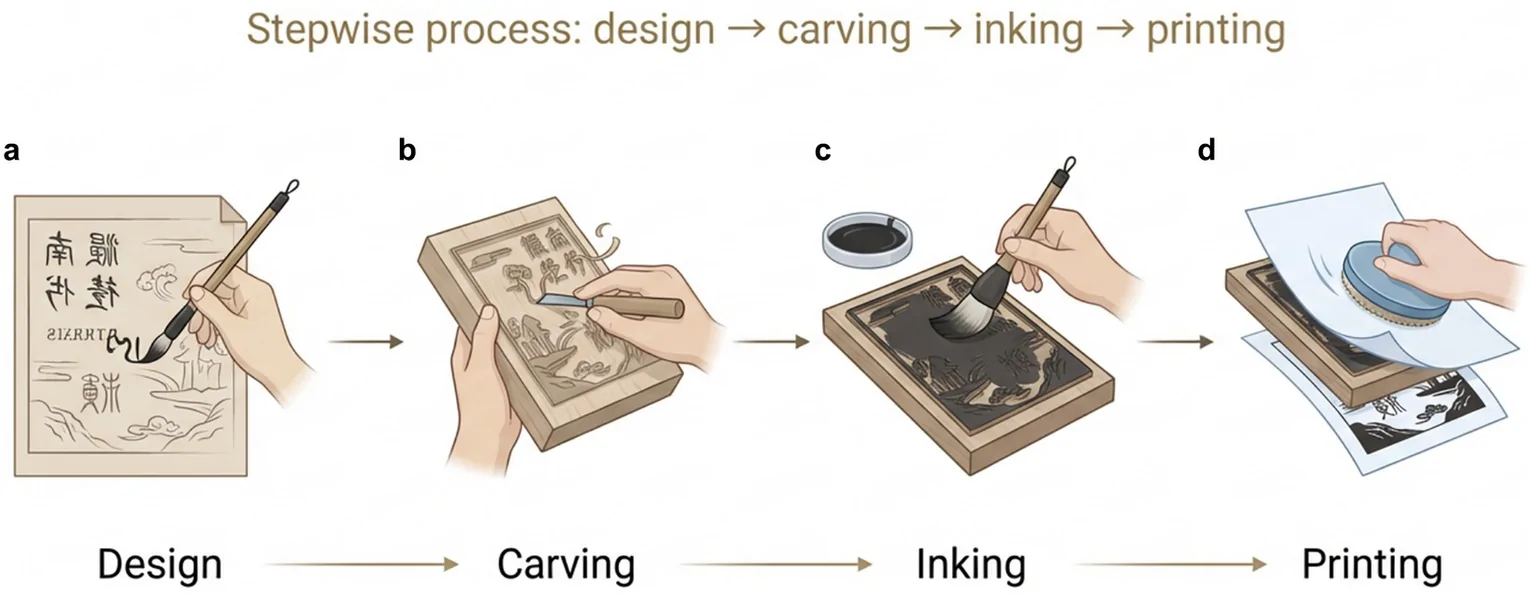
Stages of Chinese woodblock printing. Stepwise workflow illustrating the four core stages: **(a)** Design, creating a mirror-image illustration or text on paper, **(b)** Carving, cutting the design into a wooden block in raised relief, **(c)** Inking, applying ink evenly to the raised surfaces of the block, and **(d)** Printing, transferring the inked design onto paper by hand. This process integrates artistic skill with precise manual execution and forms the foundation of early mass-produced texts and images in China.

The technical process of Chinese woodblock printing is methodical and labor-intensive, integrating artistic design with precise manual execution across three core stages: design carving, ink application, and manual printing. An artist or calligrapher first creates a mirror-image design or text on thin paper, which is pasted face-down onto a polished wooden block (typically pear or jujube wood selected for its fine, dense grain). Using specialized carving knives and gouges, the artisan cuts away the non-image areas, leaving the design in raised relief, and then washes the block to remove debris. In the second stage, ink, traditionally pine-soot ink aged for optimal consistency, is evenly applied to the raised surface with a brush. Finally, a sheet of paper is laid over the inked block and gently rubbed or pressed by hand to transfer the image. Multiple impressions can be taken from a single block; for multi-color prints, separate blocks are carved and aligned with precision for successive layering (Ettinger et al., 2023; Bolwerk et al., 2014). These stages are illustrated in Figure 2 The process typically encompasses 20–30 sub-procedures, demanding both technical skill and artistic sensitivity (Li et al., 2024).

### 2.3 Motor and cognitive engagement in printmaking

The practice requires fine motor control, spatial planning, and repetitive tactile interaction, characteristics that suggest potential relevance for neurorehabilitation. Carving demands sustained precision grip, bilateral hand coordination, and controlled pressure to execute intricate lines without error. Inking and printing involve rhythmic, repetitive upper-limb movements and tactile feedback from brush pressure and paper manipulation, while the overall workflow necessitates visuospatial planning to account for mirror-image reversal, proportional scaling, and multi-block registration in color printing. These sensorimotor and cognitive demands engage executive functions such as sequencing, attention to detail, and problem-solving (Bai et al., 2021). Table 1 presents the specific motor and cognitive skills engaged at each stage.

**Table 1:** Cognitive and motor skills engaged in printmaking.

Process step	Motor demand	Cognitive function	Ref(s)
Design carving	Sustained precision grip, bilateral hand coordination, and finely controlled pressure to execute intricate lines and details without error	Advanced visuospatial planning, including accurate mirror-image reversal and proportional scaling of the original design onto the wooden block	Saggar et al. (2015)
Ink application	Rhythmic, repetitive upper-limb movements using a brush to apply pine-soot ink evenly across the raised relief surface	Continuous attention to detail and real-time tactile feedback to maintain uniform ink distribution and pressure consistency	Chen et al. (2020), Arkin et al. (2019), and Fauvel et al. (2014)
Manual printing	Gentle pressing and rubbing of paper onto the inked block, engaging repetitive upper-limb movements together with fine tactile manipulation and hand-eye coordination	Precise spatial alignment and registration of the paper to ensure accurate image transfer, especially during multi-color layering	Andrews et al. (2021) and Minutillo et al. (2021)
Overall printmaking workflow	Repetitive sensorimotor tasks across 20–30 sub-procedures, combining fine motor control, bilateral coordination, and sustained upper-limb activity throughout the entire process	Core executive functions such as sequencing, problem-solving, sustained attention to detail, and holistic visuospatial integration across all stages	Liebscher et al. (2023) and Bailey et al. (2014)

## 3 Neuroscience of art and neuroplasticity

### 3.1 Neural mechanisms of creative activity

Artistic engagement activates a sophisticated topography of brain regions, primarily encompassing the motor, prefrontal, and parietal cortices. Functional magnetic resonance imaging (fMRI) and transcranial magnetic stimulation (TMS) studies indicate that creative motor tasks recruit a distributed fronto-parieto-motor network (Saggar et al., 2015; Martel and Glover, 2023). Within this architecture, the primary motor cortex and supplementary motor area govern the execution and sequencing of the precise manual movements essential for drawing, carving, or printing. Simultaneously, the dorsolateral and ventromedial prefrontal cortices support high-level executive functions, including strategic planning, decision-making, and creative problem-solving.

The superior and inferior parietal lobules facilitate visuospatial processing, attention to detail, and sensorimotor transformation, allowing for the accurate translation of mental imagery into physical action (Kowatari et al., 2008). While these activations occur bilaterally, they often exhibit right-hemisphere dominance during novel or emotionally expressive tasks, reflecting the synergy between motor control and higher-order cognition (Chen et al., 2020). Longitudinal neuroimaging further reveals that sustained artistic practice strengthens functional connectivity within this network, providing a plausible neural substrate for conceptual neurorehabilitation relevance.

### 3.2 Sensorimotor learning and plasticity

Creative motor activities stimulate synaptic plasticity and neural network remodeling. At the cellular level, artistic engagement upregulates brain-derived neurotrophic factor (BDNF) and other neurotrophins that promote dendritic spine formation, long-term potentiation, and synaptic strengthening within the motor and prefrontal cortices (Schlegel et al., 2015). Structural changes include increased gray-matter volume in the right middle frontal gyrus and enhanced white-matter integrity along sensorimotor and auditory-motor pathways, as documented in both musical training and visual art interventions (Chamberlain et al., 2014; Hong et al., 2023). These remodeling processes are activity-dependent: sustained practice induces experience-driven reorganization of cortical maps, improves motor learning efficiency, and facilitates compensatory plasticity in neurodegenerative conditions such as Parkinson’s disease (Hola et al., 2024). Network-level analyses show strengthened resting-state connectivity between the default mode network and task-positive networks, supporting the transfer of creative skills to everyday motor and cognitive functions (Barnett and Vasiu, 2024).

### 3.3 Emotional and cognitive benefits

Artistic activities reduce stress and enhance cognitive resilience. By modulating activity in the amygdala and medial prefrontal cortex, artistic engagement downregulates the hypothalamic–pituitary–adrenal axis, lowering cortisol levels and alleviating anxiety and depressive symptoms commonly associated with chronic neurological disorders (Kaimal et al., 2016). Cognitive benefits include improved executive function, working memory, and emotional regulation, mediated through repeated activation of prefrontal–parietal circuits (Ettinger et al., 2023). These effects are particularly pronounced in group-based creative practices, which additionally foster social connectedness and self-efficacy (Forgeard et al., 2021). Table 2 summarizes representative evidence linking artistic engagement and neuroplasticity-related mechanisms relevant to neurological rehabilitation.

**Table 2:** Evidence linking art practice and neuroplasticity.

Study	Art intervention	Neurological outcome	Ref(s)
fMRI and TMS studies of creative motor tasks	Drawing, carving, or printing	Artistic engagement activates a distributed fronto-parieto-motor network, including the primary motor cortex and supplementary motor area for fine hand-movement execution and sequencing, dorsolateral and ventromedial prefrontal cortices for planning and creative problem-solving, and superior/inferior parietal lobules for visuospatial processing and sensorimotor transformation; right-hemisphere dominance is observed during novel or emotionally expressive tasks.	Loui et al. (2019), Luo et al. (2014), and Zamorano et al. (2017)
Longitudinal neuroimaging of repeated artistic practice	Sustained creative motor activities	Repeated artistic practice strengthens functional connectivity within the fronto-parieto-motor network, supporting a plausible neural basis for longer-term neurorehabilitation-related effects.	Kaimal et al. (2016)
Cellular-level studies of neurotrophin expression	Artistic engagement	Creative motor activities upregulate brain-derived neurotrophic factor (BDNF) and other neurotrophins, promoting dendritic spine formation, long-term potentiation, and synaptic strengthening specifically within motor and prefrontal cortices.	Kaimal et al. (2017)
Structural neuroimaging in musical training and visual art interventions	Musical training and visual art practice	Sustained practice produces increased gray-matter volume in the right middle frontal gyrus and enhanced white-matter integrity along sensorimotor and auditory-motor pathways.	Forgeard et al. (2021) and Tong et al. (2021)
Experience-driven plasticity studies	Sustained creative motor practice	Activity-dependent cortical map reorganization occurs, improving motor learning efficiency and potentially supporting compensatory plasticity in neurodegenerative conditions such as Parkinson’s disease.	Perkins et al. (2021)
Resting-state network analyses	Creative activities	Strengthened functional connectivity is observed between the default mode network and task-positive networks, supporting the transfer of creative skills to everyday motor and cognitive functions.	Barnett and Vasiu (2024)
Stress-axis modulation studies	Artistic engagement	Art activities downregulate the hypothalamic–pituitary–adrenal axis through modulation of the amygdala and medial prefrontal cortex, lowering cortisol levels and reducing anxiety and depressive symptoms commonly associated with neurological disorders.	Strang (2024)
Cognitive circuit studies	Art activities	Repeated activation of prefrontal–parietal circuits improves executive function, working memory, and emotional regulation.	Wu et al. (2022)
Group-based creative practice studies	Group creative activities	Socially embedded artistic practices enhance self-efficacy and social connectedness in addition to the core neuroplastic benefits.	Lee and Ko (2023)

**Figure 3:**
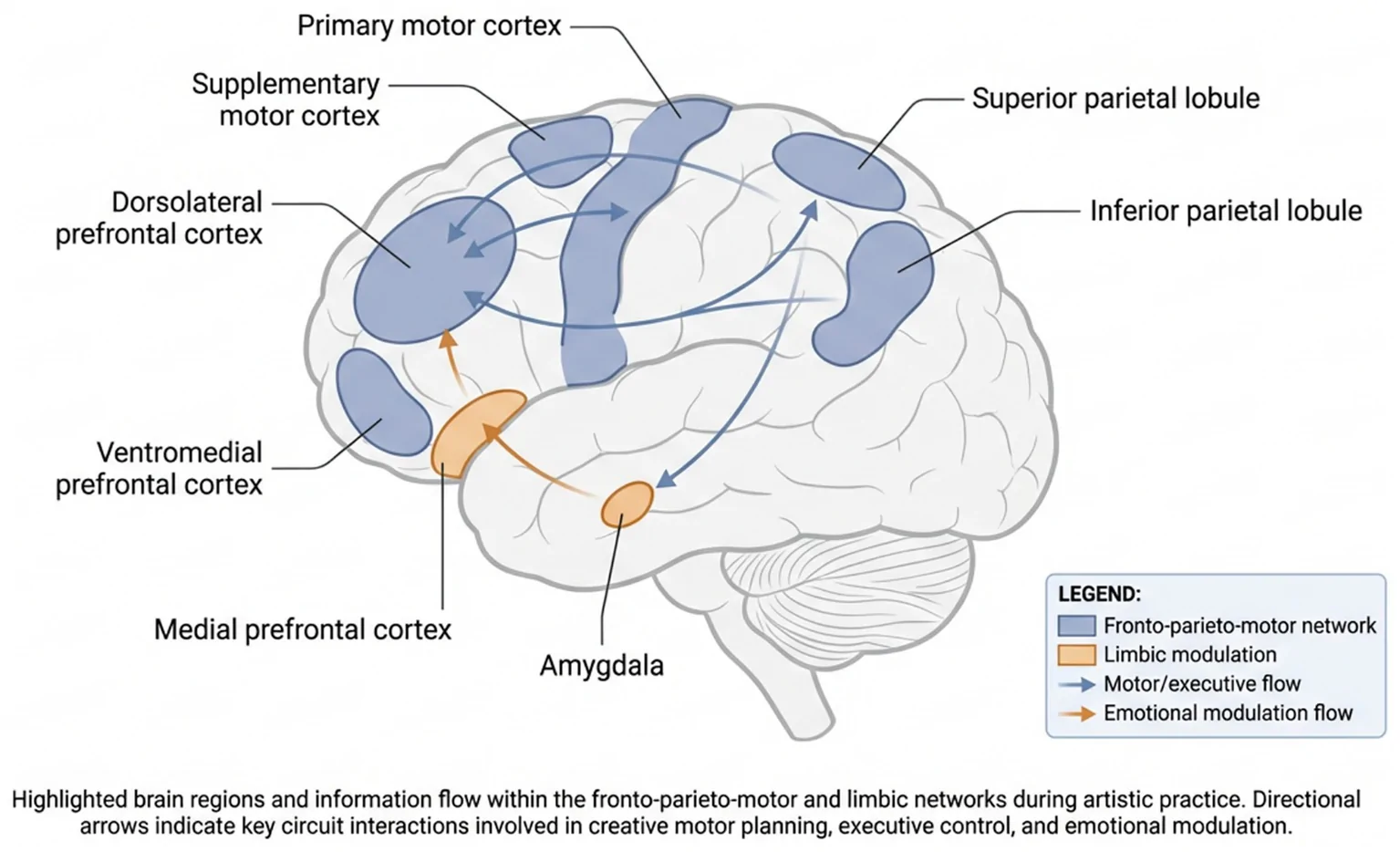
Proposed neural circuits activated during Chinese woodblock printing as a neurorehabilitation intervention. Illustration of key brain regions and interacting networks hypothesized to be engaged during artistic activity. The fronto–parieto–motor network (blue) includes the primary and supplementary motor cortices, parietal lobules, and dorsolateral prefrontal cortex, supporting motor planning, coordination, and executive control. Limbic regions (orange), including the amygdala and ventromedial/medial prefrontal cortices, contribute emotional modulation. Arrows indicate bidirectional information flow between cognitive-motor and affective systems, highlighting the integration of motor execution, executive processes, and emotional regulation during creative practice.

Collectively, these findings provide a conceptual neurobiological framework through which the structured sensorimotor demands of Chinese woodblock printing may plausibly engage rehabilitation-relevant pathways in Parkinson’s disease. Figure 3 illustrates the proposed neural substrate for Chinese woodblock printing as a culturally grounded sensorimotor intervention.

## 4 Art-based interventions in Parkinson’s disease

### 4.1 Motor rehabilitation through creative practice

Creative arts serve as potent catalysts for improving dexterity, coordination, and motor planning in Parkinson’s disease (PD). A growing literature suggests that art-based interventions were associated with reduced motor symptom severity, as measured by the Unified Parkinson’s Disease Rating Scale Part III (UPDRS-III; standardized mean difference [SMD] = −0.44, *p* < 0.05), and improved dynamic balance (Mini-BESTest SMD = 0.41, *p* < 0.05) (Liu et al., 2025).

**Figure 4:**
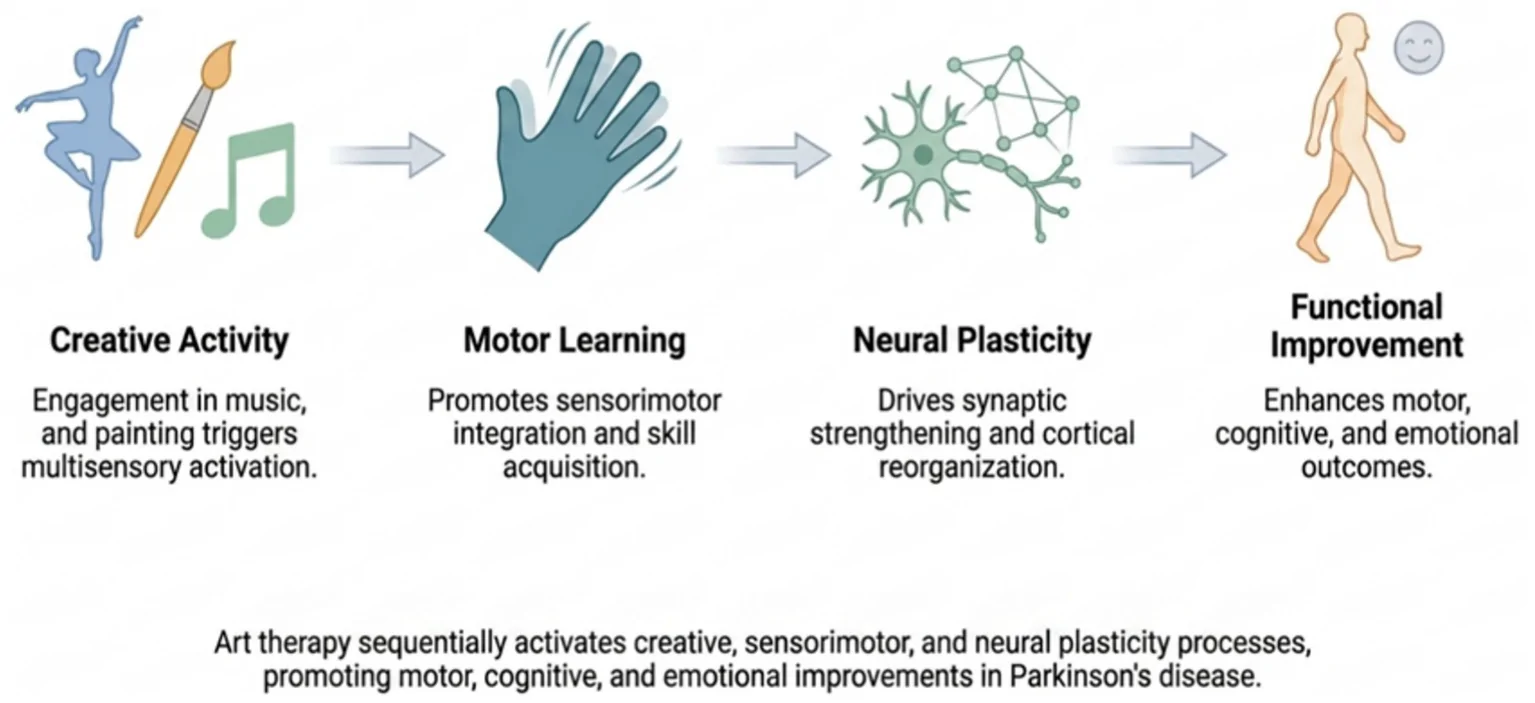
Proposed mechanisms of art-based interventions. Schematic illustrating the hypothesized processes through which art-based interventions may exert therapeutic effects in Parkinson’s disease. Engagement in creative activities, music, and painting initiates multisensory stimulation, which may facilitate motor learning through enhanced sensorimotor integration and skill acquisition. This, in turn, may promote neural plasticity, including synaptic strengthening and cortical reorganization. Together, these processes may contribute to functional improvements across motor, cognitive, and emotional domains.

Complementary practices, such as rhythmic drumming, have also shown promise for upper-extremity function. Small-scale studies report improved motor control, coordination, and dexterity following structured sessions utilizing rhythmic cueing (Park and Kim, 2021; Fan et al., 2022). These gains are attributed to the repetitive, goal-directed movements inherent in creative practice, which engage sensorimotor integration and motor planning circuits often compromised in PD. While general physiotherapy typically yields short-term benefits (<3 months) (Tomlinson et al., 2012), sustained creative interventions (≥12 weeks) appear to produce longer-lasting functional improvements (Duncan and Earhart, 2011). These underlying pathways are illustrated in Figure 4.

### 4.2 Psychological and social benefits

Beyond physical outcomes, art programs significantly enhance mood, self-expression, and social engagement. Music therapy has been shown to improve emotional function on the Happiness Measure (*p* < 0.0001) in PD cohorts (Pacchetti et al., 2000) and to reduce anxiety while increasing overall well-being (Pohl et al., 2020). Broader art-therapy approaches similarly alleviate depressive symptoms and neuropsychiatric burden, with review-level evidence indicating moderate effect sizes on emotional regulation scales (Spee et al., 2025). Both qualitative and quantitative data converge on the value of creative self-expression for restoring a sense of agency and identity—outcomes that are particularly salient given the progressive loss of autonomy associated with PD. Furthermore, group-based programs amplify social engagement by fostering peer interaction, reducing isolation, and building communal support networks, which are critical factors for long-term treatment adherence (Heiberger, 2011).

### 4.3 Global art therapy programs

Established global art therapy initiatives include diverse programs such as painting workshops, calligraphy-based practice, and music therapy. Painting and visual-art sessions are widely implemented in clinical settings across Europe, North America, and Asia, often integrated into conventional rehabilitation frameworks. Fine-motor art protocols, including calligraphy-oriented training, are increasingly used in rehabilitation research because they combine precision grip, visuospatial planning, and repetitive hand control. Similarly, music therapy programs, delivered both individually and in groups, have been adopted by specialized PD centers worldwide and are supported by evidence suggesting emotional and behavioral symptom relief (Pacchetti et al., 2000; Zhou et al., 2021). These culturally adaptable, low-cost interventions are increasingly incorporated into multidisciplinary care pathways, reflecting a paradigm shift toward holistic, patient-centered neurorehabilitation. The art-based therapies relevant to Parkinson’s disease are summarized in Table 3.

**Table 3:** Art-based therapies evaluated in Parkinson’s disease.

Therapy type	Outcome measures	Key findings	Ref(s)
General art-based interventions	UPDRS-III, Mini-BESTest	Review-level evidence suggests that art-based interventions significantly reduced motor symptom severity (UPDRS-III standardized mean difference −0.44, *p* < 0.05) and improved dynamic balance (Mini-BESTest standardized mean difference 0.41, *p* < 0.05).	Machado Sotomayor et al. (2021.)
Rhythmic drumming	Motor control, coordination, dexterity	Structured drumming sessions with rhythmic cueing improved upper-extremity motor control, coordination, and dexterity through repetitive, goal-directed sensorimotor engagement.	Duncan and Earhart (2011), and Duncan and Earhart (2014)
Music therapy	Happiness Measure, anxiety and well-being scales, Neuropsychiatric Inventory	Music therapy significantly improved emotional function (Happiness Measure, *p* < 0.0001), reduced anxiety, and increased well-being in PD patients. Broader evidence supports strong relief of emotional and behavioral symptoms when delivered individually or in groups.	Foster et al. (2013), Hackney and Earhart (2009a), and Hackney and Earhart (2009b)
Broader art therapy	Emotional regulation scales, depressive symptoms, neuropsychiatric burden	Art-therapy approaches alleviate depressive symptoms and neuropsychiatric burden, with review-level evidence showing moderate effect sizes on emotional regulation scales while restoring a sense of agency and identity.	McNeely et al. (2015)
Visual art (painting workshops)	Motor function, psychological well-being, social engagement	Painting and visual-art sessions, widely implemented in community and clinical settings across Europe, North America, and Asia, support motor rehabilitation, psychological well-being, and social connectedness when integrated with conventional neurorehabilitation.	Fontanesi and DeSouza (2021)

## 5 Chinese woodblock printing as a hypothesis-generating candidate for neurorehabilitation

### 5.1 Fine motor training

Chinese woodblock printing involves repetitive and highly structured hand movements that theoretically engage motor processes relevant to rehabilitation in Parkinson’s disease (PD), although no direct clinical studies of woodblock printing in PD currently exist. The carving phase demands sustained precision grip, controlled pressure, and bilateral coordination to incise fine lines and textures into the wooden block, while inking and printing involve rhythmic brushing and pressing motions that engage the intrinsic hand muscles and forearm extensors. Comparable fine-motor and sensorimotor demands have been examined in analogous creative interventions, including modified calligraphy exercises and rhythmic drumming paradigms, where improvements in upper-limb motor function, coordination, and dexterity were reported in stroke and PD populations (Proud et al., 2024; Vorasoot et al., 2020). However, these findings derive from those specific modalities rather than from woodblock printing itself.

Review-level evidence suggests that art-based interventions in PD may be associated with improvements in motor symptoms and balance, including reduced UPDRS-III scores and better Mini-BESTest performance (Llamas-Velasco et al., 2023). Because Chinese woodblock printing incorporates precision grip, bilateral coordination, visuospatial planning, and repetitive practice, it may theoretically engage rehabilitation-relevant mechanisms similar to those described in calligraphy, drawing, and rhythmic motor training studies. Nevertheless, no empirical evidence currently demonstrates that woodblock printing itself slows motor decline or induces compensatory neuroplastic changes in PD.

To further contextualize these indirect mechanistic parallels, Table 4 summarizes representative art-based and sensorimotor interventions that share functional similarities with traditional Chinese woodblock printing in neurological rehabilitation settings. This table presents representative peer-reviewed studies and reviews selected on the basis of mechanistic overlap with the principal sensorimotor demands of Chinese woodblock printing, specifically sustained precision grip, bilateral hand coordination, visuospatial sequencing, and repetitive upper-limb engagement. Entries were drawn from the broader fine-motor, art-therapy, and sensorimotor rehabilitation literature; no direct studies of woodblock printing in neurological populations were identified, and no formal eligibility screening was applied.

**Table 4:** Analogous art-based and sensorimotor interventions relevant to the proposed neurocognitive framework of traditional Chinese woodblock printing.

Intervention	Neurological disorder	Key outcomes	Ref(s)
Creative visual art therapy	Stroke	An RCT of 118 patients showed that creative art therapy plus physical therapy significantly enhanced overall physical function (mean difference 1.2, *p* = 0.043) compared with physical therapy alone. A scoping review of 17 studies confirmed consistent gains in limb motor function, fine motor skills, and activities of daily living.	Kongkasuwan et al. (2016) and Chan et al. (2023)
Structured sensory-motor training (creative)	Stroke	Supervised creative sensory-motor training produced >20% gains in upper-extremity function and sensory discrimination (*p* < 0.01).	Christiansen et al. (2022)
Structured creative activities	Pediatric Intellectual Disability	Structured creative activities yielded positive effects on motor planning, balance, and attention.	Kempner et al. (2024)
Mind–body and art therapies	Neurodegenerative Disorders	An RCT of 150 patients reported reductions in Difficulties in Emotion Regulation Scale scores (−4.8 vs. − 0.11 in controls), although the between-group difference was not statistically significant (*p* = 0.13).	Tsimaras et al. (2012)
Music therapy	Parkinson’s disease and dementia	Music therapy significantly improved emotional function on the Happiness Measure in PD patients (p < 0.0001) and reduced behavioral/psychiatric symptoms on the Neuropsychiatric Inventory in dementia patients (*p* = 0.002). Pilot data in PD confirmed significant anxiety reduction and well-being gains.	Pacchetti et al. (2000), Pohl et al. (2020), and Raglio et al. (2008)
Modified calligraphy exercises	Stroke	Modified calligraphy significantly improved upper-limb motor function on the Fugl-Meyer Assessment (*p* = 0.001).	Wu et al. (2022)
Art therapy (general)	Parkinson’s disease	Review-level evidence suggests improvements in motor symptoms and balance in PD.	Zhang et al. (2022)
Rhythmic drumming	Neurological disorders (including PD)	Drumming with rhythmic cueing enhanced upper-extremity motor control and attention; case-series data additionally showed favorable changes in balance and dexterity.	Park and Kim (2021) and Taghizadeh et al. (2018)
Chinese woodblock printing (proposed)	Parkinson’s disease (potential)	No direct empirical studies exist on neural activation or biomechanical effects during woodblock printing tasks. Closest evidence from structured visuospatial block-design tasks indicates increased activation in memory-, motor-, and arithmetic-related cortical regions, with broader literature highlighting parietal (superior parietal lobule), prefrontal, and visual cortical involvement, suggesting plausible sensorimotor and neuroplastic benefits.	De Pisapia et al. (2016), Aziz-Zadeh et al. (2012), Miall et al. (2009), and Lorey et al. (2011)

Collectively, these analogous interventions suggest plausible overlap in fine-motor coordination, visuospatial sequencing, repetitive sensorimotor engagement, and attentional control processes that may also be relevant to woodblock printing activities. However, these parallels should be interpreted as hypothesis-generating rather than direct evidence of clinical efficacy.

### 5.2 Multisensory stimulation

Chinese woodblock printing combines tactile perception, visual feedback, and sequential cognitive processing within a single structured activity. Carving provides immediate haptic feedback through knife resistance and wood grain texture, while inking delivers visual-tactile integration as the artist monitors ink distribution and paper adhesion. Printing further requires precise spatial alignment of successive blocks for multi-color work, demanding visuospatial processing and working memory. Studies of analogous creative motor activities, including drawing, calligraphy, and other visuomotor artistic tasks, have shown activation and remodeling of fronto-parieto-motor networks associated with sensorimotor integration and executive control (Barnett and Vasiu, 2024). Direct neuroimaging evidence specific to woodblock printing, however, has not yet been reported.

Compared with unimodal exercise paradigms, the combined tactile, visual, and sequential demands of woodblock printing may plausibly support sensorimotor integration and proprioceptive engagement, based on mechanistic parallels with enriched multisensory rehabilitation approaches described in the broader neurorehabilitation literature (Strang, 2024). However, this hypothesis has not been directly evaluated in PD populations performing woodblock printing.

The structured, stepwise nature of the workflow also trains executive functions such as task sequencing and error monitoring, with potential transfer to improvements in activities of daily living. At present, these proposed benefits remain conceptual and should be interpreted as hypothesis-generating rather than evidence of established clinical efficacy.

### 5.3 Cultural engagement and identity

Incorporating culturally meaningful art practices may enhance patient motivation, engagement, and emotional connection to rehabilitation activities. For individuals from East Asian backgrounds or those with cultural affinity for Chinese heritage, woodblock printing offers a meaningful link to ancestral artistic traditions, fostering a sense of identity and purpose that generic rehabilitation exercises often lack. Qualitative evidence from culturally tailored art therapies indicates that such interventions improve adherence, self-efficacy, and emotional well-being by aligning therapeutic activities with patients’ lived experiences and values (Spee et al., 2025). In PD, where apathy and reduced motivation are common non-motor symptoms, culturally resonant activities may improve long-term participation and adherence, as suggested by broader literature on culturally tailored creative therapies (Li et al., 2024). However, these observations have not been specifically validated for Chinese woodblock printing interventions.

**Figure 5:**
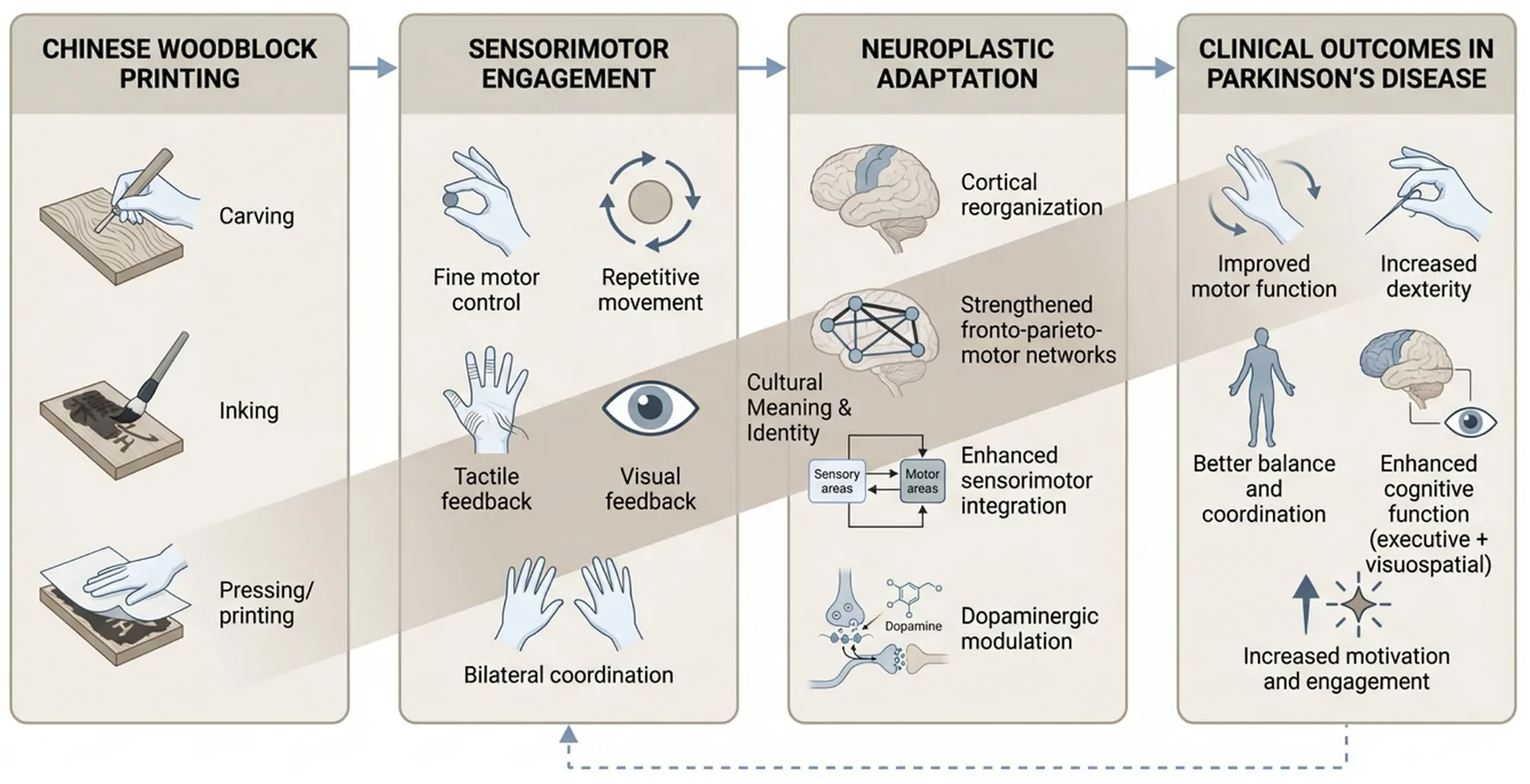
Proposed mechanisms linking Chinese woodblock printing to functional improvements in Parkinson’s disease. Conceptual framework illustrating how engagement in Chinese woodblock printing (carving, inking, and pressing/printing) may drive therapeutic effects in Parkinson’s disease. These structured artistic tasks promote sensorimotor engagement through fine motor control, repetitive movement, tactile and visual feedback, and bilateral coordination. Sustained practice is hypothesized to support neuroplastic adaptation, including cortical reorganization, strengthened fronto–parieto–motor networks, enhanced sensorimotor integration, and dopaminergic modulation, further enriched by cultural meaning and identity. Collectively, these changes may contribute to improved clinical outcomes, including enhanced motor function and dexterity, better balance and coordination, improved cognitive performance (executive and visuospatial), and increased motivation and engagement.

Figure 5 illustrates a hypothetical conceptual pathway through which the sensorimotor and cognitive demands of woodblock printing could contribute to neuroplastic adaptation and functional rehabilitation in Parkinson’s disease. This model integrates the fine-motor, multisensory, and cultural dimensions previously outlined, providing a robust and testable framework for future clinical trials.

Collectively, although direct empirical trials of Chinese woodblock printing in neurological rehabilitation remain absent, the modality shares several biomechanical and cognitive characteristics with established fine-motor and art-based interventions. These parallels justify further investigation of woodblock printing as a potential low-cost, culturally grounded adjunctive approach for PD neurorehabilitation.

## 6 Public health implications

### 6.1 Integrating cultural arts in healthcare systems

Community art programs offer a viable complement to traditional medical therapies by addressing the psychosocial and functional dimensions of recovery that conventional rehabilitation often overlooks (Stuckey and Nobel, 2010). In particular, culturally resonant fine-motor printmaking workshops such as Chinese woodblock printing can extend this logic by providing repetitive sensorimotor practice in a low-cost, accessible, and identity-affirming format. Review-level evidence of visual arts-based interventions for stroke survivors demonstrate statistically significant improvements in depressive symptoms (standardized mean difference [SMD]: −1.14), activities of daily living (SMD: 0.96), and upper-limb function (SMD: 0.83) (Chan et al., 2023). These benefits persist beyond the acute phase and enhance overall quality of life (Morris et al., 2017). Creative engagement activates neuroplastic pathways, fosters emotional expression, and rebuilds self-efficacy in ways that pharmacological or physical therapies alone cannot achieve (Barnett and Vasiu, 2024). Accordingly, the public-health value of community printmaking lies not only in symptom support but also in its potential to sustain participation, social connectedness, and culturally meaningful rehabilitation. Hospital- and community-based artist-in-residence models further embed the arts within clinical workflows, reducing anxiety, promoting social connection, and lowering healthcare costs by supporting long-term adherence to rehabilitation regimens (Li et al., 2021; Jensen et al., 2024). The same implementation logic supports community woodblock printing workshops as scalable adjuncts within rehabilitation and public-health systems.

**Figure 6:**
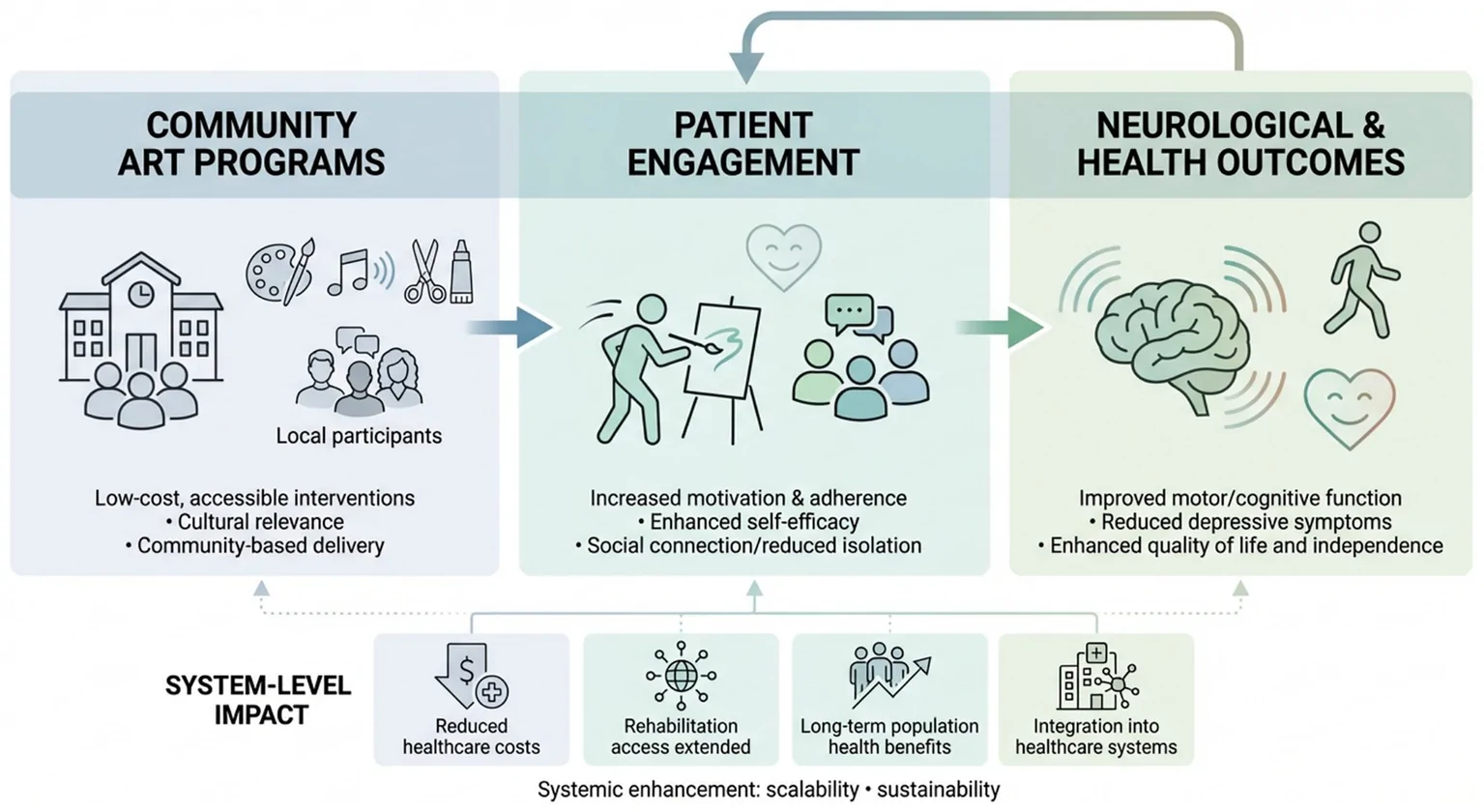
Community-based art programs and their impact on patient engagement and health outcomes. Conceptual model illustrating how community art programs may contribute to neurological and health improvements through enhanced patient engagement. Accessible, low-cost, and culturally relevant community-based interventions foster participation in creative activities, which in turn may increase motivation, adherence, self-efficacy, and social connection while reducing isolation. These engagement processes may support improvements in motor and cognitive function, alleviate depressive symptoms, and enhance overall quality of life and independence. At the system level, such programs may offer scalable and sustainable benefits, including reduced healthcare costs, expanded access to rehabilitation, long-term population health gains, and integration into healthcare systems.

The Public Health Model of Art-Based Rehabilitation, as illustrated in Figure 6, conceptualizes this synergy as an iterative pathway where community programs generate initial patient engagement, which subsequently drives measurable neurological improvements (Clift et al., 2019). Applied to woodblock printing, the model suggests that culturally resonant workshops may act as entry points into rehabilitation by lowering participation barriers and converting therapeutic practice into a socially meaningful community activity. This model underscores that participation in culturally resonant arts activities, whether visual, performative, or craft-based, serves as both a therapeutic modality and a social determinant of health, bridging the gap between clinical care and everyday lived experience (Gordon-Nesbitt and Howarth, 2019).

### 6.2 Accessibility and community-based interventions

Low-cost art programs substantially improve access to rehabilitation, particularly for underserved populations facing financial, geographic, or mobility barriers (Gallagher et al., 2024). Community-based printmaking workshops are particularly well suited to this aim because they rely on inexpensive tools and materials, can be delivered in non-specialized venues, and can be scaled for participants with varying motor capacity. By leveraging local venues, volunteer artists, and minimal materials, these initiatives deliver practical benefits without the overhead of specialized clinical infrastructure (Stickley et al., 2016). Participatory arts sessions in community settings have been shown to enhance mood, resilience, and social connectedness among individuals with chronic conditions, effectively extending the reach of formal rehabilitation services into neighborhoods and homes (Perkins et al., 2021; Perkins et al., 2022). Programs such as “Healing Arts Chicago,” which trains local artists as certified community health workers, exemplify scalable, equity-focused models that integrate creativity directly into primary care and public health delivery (Richmond-Cullen, 2018).

### 6.3 Global cultural health approaches

Integrating traditional arts into healthcare aligns with holistic public health models that recognize culture as a core determinant of well-being (Dow et al., 2023). In this framework, fine-motor printmaking workshops are not merely recreational; they are culturally legible health activities that can reinforce identity, purpose, and sustained engagement in rehabilitation. World Health Organization (WHO) guidelines on community-based rehabilitation explicitly position cultural and arts activities as essential for enabling people with disabilities to participate fully in society, thereby supporting mental, physical, and social recovery (Khasnabis and Motsch, 2008). Indigenous and non-Western traditions of music and visual storytelling embody this holistic ethos; embedding them within modern health systems respects cultural diversity while amplifying therapeutic efficacy (Tong et al., 2021). Such approaches shift public health from a purely biomedical paradigm toward one that values collective identity, resilience, and preventive wellness (Chu et al., 2018).

Collectively, these strategies position cultural arts not as ancillary enrichment but as potentially valuable, cost-conscious components of comprehensive public health policy (Meulenberg et al., 2023). Accordingly, culturally resonant fine-motor printmaking workshops, including Chinese woodblock printing, should be considered a plausible community-based rehabilitation model rather than an optional add-on. Future implementation research should quantify long-term cost savings and population-level neurological outcomes to guide scalable adoption worldwide (Golden et al., 2024).

## 7 Research gaps and future directions

### 7.1 Limited clinical trials

Despite growing evidence supporting visual arts-based interventions in stroke rehabilitation, few studies have directly evaluated printmaking as a targeted therapeutic modality for neurological disorders (Liu et al., 2024). Although visual arts interventions have been associated with improvements in depressive symptoms, upper-limb function, and participation, printmaking is rarely isolated as a distinct therapeutic modality (Kongkasuwan et al., 2016). Dedicated clinical trials examining printmaking-specific techniques, such as relief printing or screen printing, remain scarce; the existing literature is largely dominated by small-scale pilots, feasibility studies, and qualitative explorations rather than rigorous randomized controlled trials (Yun et al., 2023).

This paucity of targeted research is particularly pronounced in neurological populations, where the repetitive, bilateral hand movements and cognitive sequencing inherent to printmaking could uniquely stimulate neuroplasticity in motor and visuospatial networks (Morris et al., 2017). Broader creative arts reviews underscore that printmaking’s therapeutic mechanisms, including tactile feedback, sequential planning, and error-tolerant creativity, have not been systematically quantified against conventional rehabilitation (Ellis-Hill et al., 2019). Consequently, clinicians lack standardized guidelines regarding optimal protocols, dosage, or patient selection, constraining the evidence-based integration of this low-cost intervention into neurological care pathways (Pang et al., 2021).

### 7.2 Need for interdisciplinary research

Future studies must bridge disciplinary silos by integrating neuroscience, public health, and art therapy perspectives. Neuroscience can leverage neuroimaging, such as functional magnetic resonance imaging (fMRI) and electroencephalography (EEG), to map printmaking-induced neuroplastic changes in sensorimotor and prefrontal networks, elucidating mechanisms beyond those observed in generic visual arts (Strang, 2024). Public health frameworks are necessary to evaluate scalability, cost-effectiveness, and equity in community-based delivery, ensuring interventions reach underserved populations facing mobility or geographic barriers (Barnett and Vasiu, 2024). Art therapy expertise is essential for standardizing protocols, training facilitators, and incorporating culturally responsive practices (Reitere et al., 2024). Such collaboration would generate robust, translatable evidence that aligns printmaking with holistic rehabilitation models.

### 7.3 Longitudinal outcome studies

Long-term evaluation of neuroplastic effects remains essential for clinical validation. Most existing trials assess outcomes immediately post-intervention, leaving unanswered questions regarding the durability of gains in motor function, cognitive resilience, and quality of life (Cucca et al., 2018). The next translational step should be a staged program of woodblock-printing-specific research. First, feasibility and acceptability studies should establish whether standardized sessions can be delivered safely and consistently in outpatient, community-based, or home-supported rehabilitation settings, and should define practical parameters such as session length, therapist training, materials, and dose. Second, pilot neurorehabilitation trials should compare woodblock printing with active controls and assess motor-dexterity outcomes, including finger dexterity, hand function, bilateral coordination, and bradykinesia, together with visuospatial cognition, executive sequencing, and patient-reported fatigue or burden. Third, adherence studies should quantify attendance, home-practice completion, and barriers to participation, with attention to age, disease severity, and cultural familiarity. Fourth, culturally adapted intervention protocols should be co-developed with patients, rehabilitation specialists, and art practitioners to standardize instruction, progression, and fidelity monitoring. Longitudinal follow-up over 12–24 months, ideally paired with objective biomarkers and validated clinical outcomes, will be necessary to determine durability and clinical relevance (Ettinger et al., 2023).

## 8 Conclusion

Chinese woodblock printing may represent a culturally embedded neurorehabilitation modality that plausibly bridges historical artistic practice with contemporary neuroscience. By combining repetitive fine-motor demands, multisensory stimulation, executive sequencing, and cultural resonance, the technique could engage fronto-parieto-motor and limbic networks potentially compromised in Parkinson’s disease. Evidence from analogous creative interventions, most notably music, calligraphy, and fine-motor visual art practices, suggests possible improvements in motor function, balance, emotional regulation, and quality of life, though direct trials of woodblock printing are lacking. The absence of direct trials on woodblock printing itself represents a translational gap rather than a limitation of the approach; the biomechanical and neural mechanisms align with those observed in other art-based interventions, suggesting plausible sensorimotor and neuroplastic benefits.

Beyond individual clinical gains, the public-health implications are substantial. Low-cost, community-delivered printmaking workshops could feasibly reduce healthcare expenditure, overcome geographic and mobility barriers, and address psychosocial domains (apathy, isolation, loss of identity) that standard rehabilitation often neglects. Culturally tailored programs may be particularly motivating for East Asian or heritage-connected patients, potentially enhancing adherence and engagement.

Future research should focus on hypothesis testing via well-designed RCTs comparing woodblock printing with active controls, incorporate objective neuroimaging (fMRI, EEG) and biomechanical motion-capture to map neuroplastic changes, and track longitudinal outcomes up to 12–24 months using validated PD-specific instruments (UPDRS-III, Mini-BESTest, PDQ-39). Interdisciplinary collaboration, merging neuroscience, art therapy, rehabilitation science, and public health, is warranted to establish protocols, optimize dosage, and evaluate feasibility, cost-effectiveness, and equity.

In conclusion, this narrative review and conceptual perspective positions Chinese woodblock printing as a plausible, culturally embedded neurorehabilitation concept rather than an established therapy for Parkinson’s disease. Its principal contribution is to confirm the present evidence gap and to synthesize mechanistically analogous fine-motor arts-based interventions into a testable conceptual framework centered on repetitive sensorimotor engagement, executive sequencing, and neuroplastic adaptation. Well-designed randomized trials, supported by neuroimaging and biomechanical measures, are now required to determine whether this traditional practice can translate into clinically meaningful gains in dexterity, balance, cognition, and quality of life.
